# Synthesis and Performance Evaluation of Metallocene Polyalphaolefins (mPAO) Base Oil with Anti-Friction and Anti-Wear Properties

**DOI:** 10.3390/polym16192828

**Published:** 2024-10-06

**Authors:** Qidi Hu, Kai Zeng, Sheng Han, Jian Xu, Wenjing Hu, Jiusheng Li

**Affiliations:** 1 Key Laboratory for Green Processing of Chemical Engineering of Xinjiang Bingtuan, School of Chemistry and Chemical Engineering, Shihezi University, Shihezi 832004, China; huqidi2024@sari.ac.cn (Q.H.); hansheng654321@sina.com (S.H.); 2 Laboratory for Advanced Lubricating Materials, Shanghai Advanced Research Institute, Chinese Academy of Sciences, Shanghai 201210, China; zengk@sari.ac.cn

**Keywords:** metallocene polyalphaolefin, triphenyl thiophosphate, alkylation, chemical modification, oxi-dation stability, anti-wear, extreme pressure

## Abstract

Anti-wear and anti-oxidation abilities are two key properties of lubricants that play a crucial role in ensuring long-term stable equipment operation. In this study, we aimed to develop a base oil with good anti-oxidation and anti-wear properties for use under extreme pressure. The as-prepared metallocene polyalphaolefin (mPAO) was chemically modified using the trifluoromethanesulfonic acid (TfOH) catalysis through an alkylating reaction with triphenyl phosphorothioate (TPPT). During the experiments, when the reaction temperature exceeded 70 °C or the concentration of TfOH exceeded 2.67%, the β-scission reaction in the alkylation process became significantly more pronounced. The physical and chemical properties of TPPT-modified mPAO (T-mPAO) were evaluated by nuclear magnetic resonance spectroscopy, Fourier trans-form infrared spectroscopy, gel–permeation chromatography, and ASTM standards. T-mPAO showed significantly improved antioxidant capacity, with the initial oxidation temperature increasing by 32 °C compared to the base oil, and it exhibited the slowest increase in acid number in the 96-h oven oxidation test. The tribological tests showed that T-mPAO had the lowest friction coefficient, wear track, and wear rate (72.7% lower than that of mPAO) as well as the highest P_*B*_ (238 kg) and P_*D*_ (250 kg) among all tested samples. Compared to mPAO, the average friction coefficient of TPPT-modified mPAO in the four-ball friction test was reduced by 30.5%, and by 16.4% in the TE77 reciprocating friction test. Based on the experimental results, T-mPAO had strong anti-oxidation ability and excellent lubricating performance.The successful synthesis of multifunctional mPAO has enabled lubricant base oil additization, making it possible to use it in more demanding work scenarios, greatly broadening its application scope and making lubricant formulation blending more flexible.

## 1. Introduction

Lubrication is essential for minimizing wear and friction while also prolonging the lifespan of contacting surfaces [1,2]. Effective lubrication can reduce friction and wear, prevent sintering, improve mechanical efficiency, reduce energy consumption, and extend machinery service life [3]. Lubricating oil generally consists of a base oil and additives, including extreme pressure additives, anti-wear additives, antioxidants, and viscosity index improvers. These additives maintain and enhance the base oil performance while compensating for deficiencies [4,5]. With advances in production technology, the demands for mechanical equipment with good operational performance under heavy loads, high speeds, and high temperatures have progressively increased [6]. In scenarios where fluid lubrication cannot be established, boundary lubrication typically prevails. In this case, the reduced oil film thickness between the two friction surfaces causes protrusions on the respective surfaces to engage with each other, which results in the localized elevation of temperature and pressure. At this stage, the active elements of the extreme pressure and anti-wear additives of the lubricant react with the metal to form solid protection films. To prevent catastrophic damage to equipment and economic loss from metal-to-metal adhesive wear or seizing, the requirements for extreme pressure and anti-wear additives have also been raised [7,8].

Traditional extreme pressure and anti-wear additives, such as sulfurized isobutylene, dialkyl dithiophosphate zinc, and other sulfur- and phosphorus-containing additives that produce sulfate ash, are often used in lubricating oils for their outstanding tribological properties [9]. However, the research and application of these additives are strictly limited by current emission regulations. Therefore, it is particularly important to develop new additives with lower or no sulfur, phosphorus, and sulfate ash levels. Triphenyl phosphite (TPPT) is a typical ashless extreme pressure and anti-wear additive widely used in turbine, gear, bearing, and hydraulic transmission oils. The reactivity of TPPT and alkylated TPPT with metal surfaces and their thermal degradation reactions in base oils have been extensively studied [10,11]. Mangolini et al.’s studies indicated that alkylated triphenyl thiophosphate is beneficial in enhancing the reactivity and antioxidant properties of the lubricant [12]. Polyalphaolefins (PAO) is widely used in the lubrication due to its better oxidation and low-temperature properties [13,14]. However, the solubility of TPPT in PAO is relatively low, partly due to its high polarity, and partly because PAO possesses a regular structure and consists of isomeric hydrocarbons without heteroatoms, which restricts the solubility of certain highly polar additives. In order to increase its solubility, alkylnaphthalene or synthetic esters is usually added to assist solubilization, which increases the complexity and cost of the lubricant formulation. Therefore, increasing the solubility of high-polarity additives in PAO and enhancing PAO’s sensitivity to additives is an issue that researchers need to concern.

To increase the solubility of high-polarity additives in base oil and enhance the performance of lubricating oils in response to increasingly demanding operating conditions, researchers generally focused on improving the molecular structure of widely used additives or developing new additives with novel structures [15]. However, there have been limited publications on modifying the chemical structure of the base oil to enhance its molecular configuration and optimize its performance for specific applications [16]. The direct alkylation of the highly reactive benzene ring in the TPPT molecule with the base oil presents a viable approach for producing a base oil with specific anti-oxidation and anti-wear properties under extreme pressures. Potential base oils include three types of hydrotreated base oils and PAO, which have been hydrotreated to improve their stability but lack reactivity [17]. Compared to traditional PAO, metallocene polyalphaolefin (mPAO) has garnered increasing attention due to its higher viscosity index, excellent low-temperature performance, and superior shear stability. In the synthesis of mPAO using a metallocene catalyst, most chain-ending reactions occur via β-H transfer elimination, creating a significant amount of terminal alkenes and easily accessible unsaturated double bonds in metallocene polyalphaolefin (mPAO) [18]. These unsaturated double bonds readily undergo alkylation with aromatic compounds, enabling them to be grafted onto the long alkyl chains of mPAO, imparting mPAO with anti-friction and extreme pressure anti-wear properties similar to those of TPPT. This facilitates functionalization of the base oil. In addition, modification of high viscosity mPAO with TPPT also avoids expensive and hazardous hydrogenation in PAO production. Moreover, the introduction of a polar group increases the polarity of the nonpolar PAO base oil, enhancing additive solubility and easing lubricating oil formulation. Finally, functionalized base oils make the development of lubricant formulations much simpler and more flexible.

In this study, high-viscosity mPAO was alkylated with TPPT using trifluoromethanesulfonic acid (TfOH) as a catalyst to prepare a base oil with anti-oxidant and anti-wear properties even under extreme pressures. The structure of the alkylated TPPT was analyzed by nuclear magnetic resonance (NMR), Fourier transform infrared (FT-IR) spectroscopy, and gel permeation chromatography (GPC). Its thermal oxidative ability was then evaluated by pressurized differential scanning calorimetry (PDSC), thermogravimetric analysis (TGA), and oven oxidation test. The tribological properties of the alkylated TPPT were characterized using a four-ball friction tester and a TE77 reciprocating friction tester.

## 2. Materials and Methods

### 2.1. Materials

Rac-ethylenebis(1-indenyl)zirconium dichloride (CAS: 100080-82-8, >94.0%) methanol (CAS: 67-56-1, 99.0%), triisobutyl aluminum (CAS: 100-99-2, 1 M toluene solution), N,N-dimethylanilinium and tetrais(pentafluorophenyl)boeate (CAS: 118612-00-3, 97.0%) were purchased from Aladdin Biochemical Technology Co., Ltd. (Shanghai, China). Triphenyl phosphorothioate (CAS: 597-82-0, 98.0%) and trifluoromethanesulfonic acid (CAS: 1493-13-6, 98.0%) were purchased from Adamas Chemical Reagent Co., Ltd. (Shanghai, China). All reagents were utilized as received, without any additional purification. 1-Octene (CAS: 111-66-0, 97.0%) was purchased from Shanghai Qicheng Industrial Co., Ltd. (Shanghai, China) and dried with a 13× molecular sieve before use.

### 2.2. Synthesis of Metallocene PolyalphaOlefins (mPAO)

The polymerization reaction was conducted in a 500-mL flask that was first evacuated and then backfilled with high-purity nitrogen 3–4 times. Initially, 250 g of dried 1-octene (pretreated with a 13 × molecular sieve) was introduced, and the temperature was elevated to 110 °C. Subsequently, 4.2 mg of rac-ethylenebis(1-indenyl)zirconium dichloride and 9.0 mg of N,N-dimethylaniline tetra(pentafluorophenyl)borate were added to a Schlenk flask containing 5 mL of toluene solvent. Subsequently, 2 mL of a 1 M triisobutylaluminum toluene solution was added and vigorously shaken. The prepared catalyst solution was then injected into the 1-octene using a syringe to initiate the reaction while maintaining a controlled temperature of 110–120 °C. After 2 h, 1 wt% clay was introduced and stirred for an additional hour. The crude product was filtered, and unreacted monomers, solvent, and light fractions were removed under vacuum at 210 °C and 1.2 mbar using a molecular distillation apparatus. The resulting mPAO served as the raw material for subsequent alkylation reactions.

### 2.3. Alkylation of TPPT with mPAO

100 mL of petroleum ether and 11.6 g of 1-butyl trimethyl imidazole were introduced into a 250 mL three-neck flask and purged with high-purity nitrogen gas three times. The reaction temperature was subsequently lowered to −20 °C, and 17.6 g of anhydrous aluminum chloride was gradually added in three portions while maintaining constant stirring for a duration of 3 h. Following phase separation, the product was utilized without further purification.

### 2.4. Physicochemical Properties

The ASTM D2270 [19], D445 [20], D92 [21], D5950 [22], D1159 [23], D611 [24], and D664(A) [25] standards were respectively used to test the viscosity index, kinematic viscosity, flash point, pour point, acid number, aniline point, and bromine number of the modified mPAO base oil.

### 2.5. Structural Composition Analysis

The FT-IR spectra were acquired using a Spectrum Two FT-IR spectrometer (ATR; PerkinElmer, Waltham, MA, USA). Gel permeation chromatography (GPC) was employed to characterize the molecular weight distribution of the modified mPAO base oil on a Viscotek GPCmax system (Malvern Panalytical, Worcestershire, UK). The NMR spectra were recorded on a BRUKER AVANCE III HD spectrometer (Shanghai, China), operated at 400.17 (1H) and 100.62 (13C) MHz. The sulfur content and phosphorus content in the modified mPAO were determined according to ASTM D2622 [26] and ASTM D6481 [27] standards, respectively.

### 2.6. Oxidation and Thermal Stability

The initial oxidation temperature (IOT) of the modified mPAO was tested with a PDSC system (METTLER TOLEDO, Greifensee, Switzerland) and further evaluated using a self-built oven oxidation test. Briefly, 200 mL of oil was heated to 150 °C with 5 g of copper wire and a steel ball in a 250-mL beaker placed in an oven. Samples were taken every 24 h and analyzed for changes in the acid number, which indicates the oxidation ability of the oil. An SDT Q600 thermal analyzer (TA Instruments, Newcastle, PA, USA) was used to monitor the weight changes of 50 mg samples heated to 500 °C at 10 °C/min in a nitrogen atmosphere.

### 2.7. Friction and Wear Test

**Figure 1:**
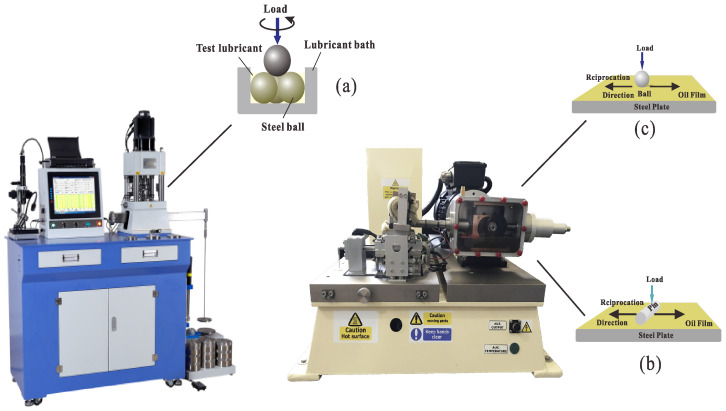
Schematic diagrams: (**a**) point-to-point, (**b**) TE77 line-on-flat, and (**c**) TE77 point-on-flat contact modes.

The tribological performance of the modified mPAO was assessed using a four-ball friction tester (Tianji Automation Co., Ltd., Xiamen, China) under point-to-point contact (Figure 1). The anti-wear performance of both samples was evaluated according to the SH/T 0189 method. The experiments were carried out at 75 °C for 60 min with a rotational speed of 1200 rpm and a load of 196 N. In the friction test, the steel balls used were manufactured by Falex Company (Chicago, IL, USA), with an average hardness of 66.1 HRC and a diameter of 12.7 mm. Subsequently, the wear mark diameter was observed by scanning electron microscopy (SEM, Mira3 XH, Tescan, Brno, Czech). The TE77 reciprocating friction tester was used to evaluate the tribological performance of mPAO, A-mPAO, and T-mPAO in the point-on-flat and line-on-flat contact modes (Figure 1). In these tests, 8-mm steel balls and a fixed steel plate (GCr15 bearing steel) were subjected to point-on-flat contact friction experiments at 75 °C for 60 min under a load of 392 N and a reciprocating frequency of 5 Hz. Additionally, a cylindrical pin (diameter 6 mm, length 16 mm) was used to conduct the line-on-flat contact friction experiment with a fixed steel plate at 75 °C for 60 min under a load of 392 N with a reciprocating frequency of 5 Hz. X-ray photoelectron spectroscopy (XPS, Kratos, AXIS Ultra DLD multifunctional) was applied to analyze the chemical state of various elements on the worn surface. In addition, the wear volume of the steel plate was measured using white light interferometry (WLI, Contour GT, Bruker, Billerica, MA, USA) and the wear rate was calculated.

## 3. Results

### 3.1. Alkylation of Trithiophenyl Phosphate and mPAO

The concentration of the catalyst, the temperature of the reaction, and the duration of the reaction affected the alkylation products of mPAO and TPPT, altering the sulfur content, viscosity, and molecular weight distribution parameters of the products, among other characteristics. By studying the reaction environment, the optimal reaction conditions can be identified to maintain the antioxidant and wear-resistant properties of the modified product, providing strong support for subsequent research. Before the alkylation reaction, unhydrogenated mPAO with a viscosity of 150 mm^2^/s at 100 °C was prepared using a metallocene catalyst and 1-octene as a raw material.

#### 3.1.1. Catalyst Dosage

**Figure 2:**
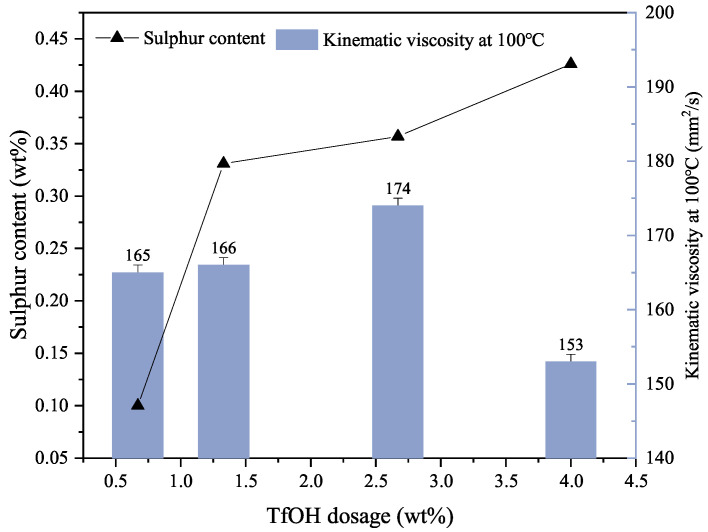
Effect of catalyst amount on triphenyl phosphorothioate alkylation with metallocene polyalphaolefin (Reaction conditions: T = 90 °C, *t* = 12 h).

The impact of catalyst quantity on the TPPT and mPAO alkylation reactions was investigated under the catalytic influence of TfOH (Figure 2). The amount of the catalyst added had a significant impact on the sulfur content and viscosity of the modified mPAO product. The sulfur content increased gradually from 0.1% to 0.426% as the catalyst amount increased. The viscosity of the product first increased with the catalyst dosage, reaching a maximum of 174 mm^2^/s at 100 °C, but decreased rapidly when the catalyst dosage exceeded 2.67%. When the amount of catalyst is increased, more Brønsted acid was added to the alkylation reaction of TPPT and mPAO, resulted in the increased sulfur content of the product. However, a higher acidity would provide positively charged protons to attack the vinylene double bond in mPAO, resulting in a more stable tri-coordinated tertiary carbon cation. This cation can attack the π bond of the phenyl ring on TPPT, forming an unstable π complex, which would then initiate alkylation reactions or undergo β-scission to produce two olefins [28]. More alkylation reactions would then occur, leading to an increase in the sulfur content of the product and a decrease in viscosity.

#### 3.1.2. Reaction Temperature

**Figure 3:**
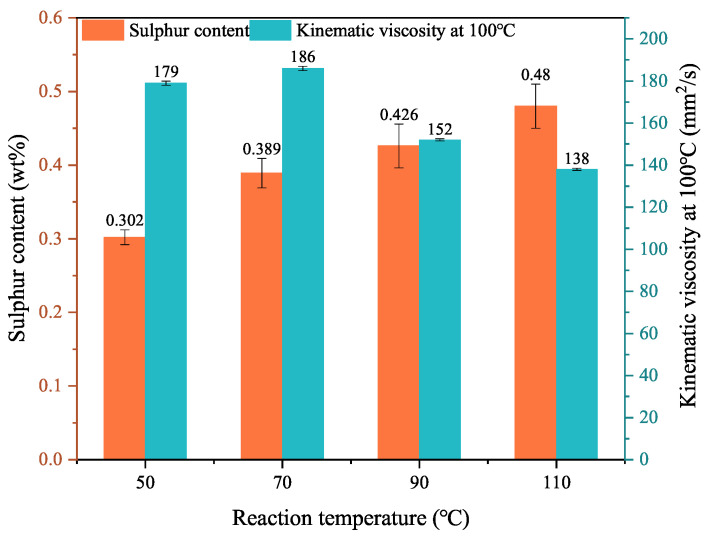
Effect of reaction temperature on triphenyl phosphorothioate alkylation with metallocene polyalphaolefin (Reaction conditions: catalyst dosage = 2.67%, *t* = 12 h).

The effect of reaction temperature on the TPPT and mPAO alkylation reactions is shown in Figure 3. The alkylation reaction began at a reaction temperature of 50 °C, and the sulfur content in the product increased from 0.3% to 0.48% as the temperature increased. Meanwhile, the viscosity of the product first increased with the reaction temperature and then decreased significantly when the reaction temperature exceeded 70 °C. When the reaction temperature was 110 °C, the kinematic viscosity at 100 °C decreased to 138 mm^2^/s, even lower than that of the reaction raw material (150 mm^2^/s). This indicated that raising the reaction temperature was generally beneficial for the alkylation reactions. As the temperature increased, the cracking reaction gradually increased, producing smaller alkenes with less steric hindrance to facilitate the alkylation reaction. However, excessive cracking reactions can result in a broader molecular weight distribution and poorer thermal stability of the base oil. Therefore, the reaction temperature should be controlled at no higher than 90 °C to reduce the occurrence of cracking reactions and ensure that the product has a certain level of sulfur content.

#### 3.1.3. Reaction Time

**Figure 4:**
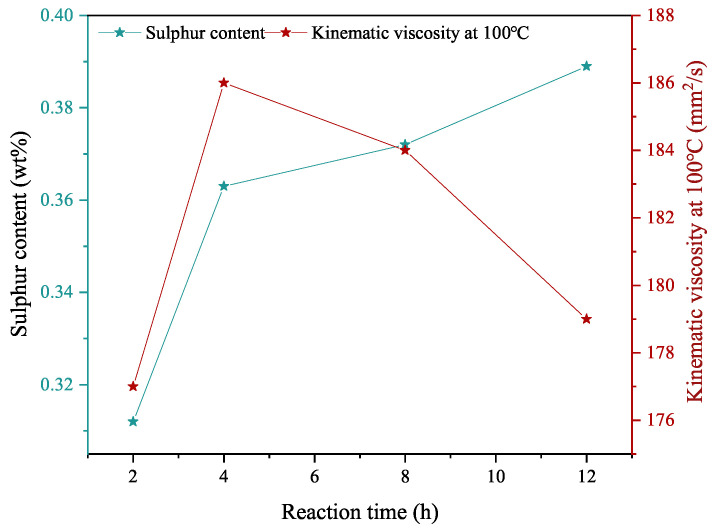
Effect of reaction time on triphenyl phosphorothioate alkylation with metallocene polyalphaolefin (Reaction conditions: catalyst dosage = 2.67%, T = 90 °C).

The effect of reaction time on the TPPT and mPAO alkylate reactions is illustrated in Figure 4. After 2 h of reaction, the sulfur content in the product reached 0.31%, while extending the reaction time to 12 h led to a maximum sulfur content of 0.39%. The viscosity of the product began to gradually decrease after 4 h, indicating a slight amount of cracking reactions during this process. After 12 h, the viscosity decreased from its peak of 186 mm^2^/s to 179 mm^2^/s at 100 °C.

### 3.2. Catalytic Mechanism

**Scheme 1:**
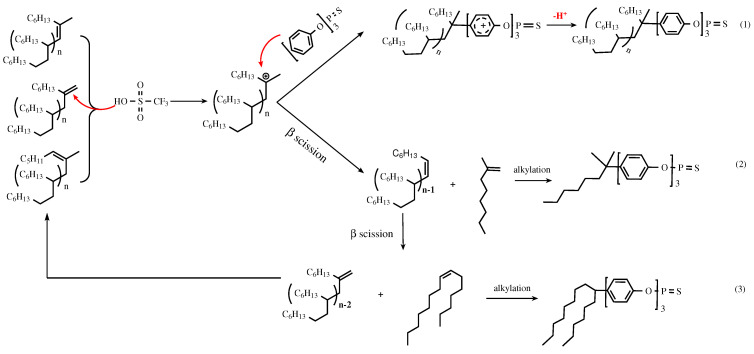
Proposed reaction route for the synthesis of TPPT-modified mPAO using TfOH. (1) Alkylation of mPAO; (2) β-scission and alkylation; (3) second β-scission and alkylation.

Based on the catalytic reaction, a possible alkylation reaction mechanism of TPPT and mPAO catalyzed by trifluoromethanesulfonic acid was proposed (Scheme 1). In the mPAO molecule, three unsaturated olefins (vinylidene, vinylene, and tri-substituted vinylene) form a tri-coordinated carbon cation in the presence of trifluoromethanesulfonic acid, which attacks the π bond at the para position of the excess TPPT phenyl ring to form an unstable π complex. The stable σ complex is then formed through carbon positive ion transfer. After H^+^ dissociation, the alkylated TPPT was produced. Increasing the reaction temperature or increasing the acid strength will cause the tertiary carbon cation to undergo β-scission [29], generating 2-methyl-1-octene and a vinylene oligomer with a lower degree of polymerization. Due to the significant reduction in steric hindrance, 2-methyl-1-octene readily undergoes alkylation with TPPT to produce 2-methyl-octyl-substituted TPPT (2). In the presence of H^+^, the vinylene oligomer undergoes further β-cleavage to generate the vinylidene oligomer with a degree of polymerization two units lower and long straight-chain alkadienes. The straight-chain alkenes are then protonated and react with TPPT to form alkylated products (3).

According to the evaluation results, increasing the reaction temperature will promote β-scission, increasing the yield of small molecule olefins and the proportion of alkylation products. This phenomenon was verified by the continuous increase in the sulfur content of the products. Similarly, increasing the catalyst concentration will greatly increase the number of catalytic active centers, producing more carbon cations and greatly increasing the probability of β-scission. However, excessive cracking reactions can produce large amounts of small molecules, which significantly reduce the flash point of the base oil and increase the complexity of the composition, resulting in a decline in thermal stability [30]. Through the appropriate control of reaction conditions, it is possible to inhibit β-scission while enhancing the sulfur content and alkylation degree, thereby leading to the improved anti-oxidation and tribological performance of the base oil.

### 3.3. Physicochemical Characterization

**Figure 5:**
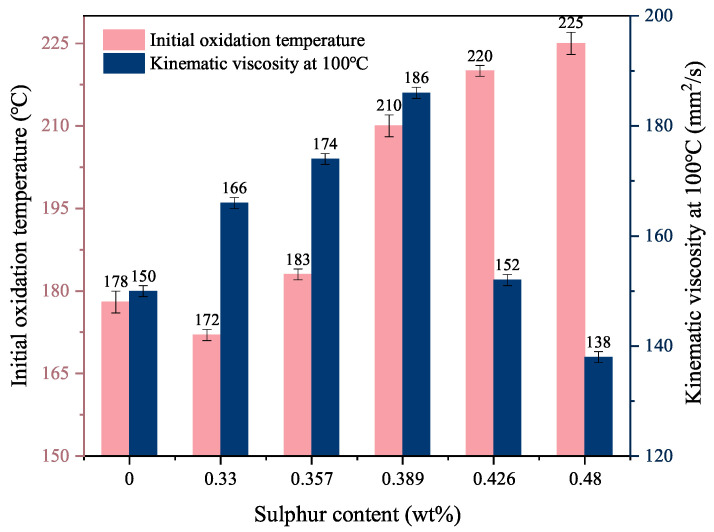
Effect of sulfur content on the initial oxidation temperature and viscosity at 100 °C of T-mPAO).

Experimental data revealed that reaction conditions can influence sulfur content in the reaction products, which may affect the performance of the modified base oil. The initial oxidation temperature (IOT) of the product was initially evaluated by PDSC, and the IOTs of TPPT-modified mPAO at different sulfur content were compared (Figure 5). The IOT increased with increasing sulfur content, from 178 °C for the mPAO raw material to a maximum of 225 °C, indicating that increasing the sulfur content can enhance the antioxidant properties of the product. The viscosity at 100 °C of the product reached a peak value of 186 mm^2^/s at a sulfur content of 0.389%. Further increasing the sulfur content caused the viscosity at 100 °C to significantly decrease to a minimum of 138 mm^2^/s, even lower than that of the raw material (150 mm^2^/s). As the sulfur content increased, the occurrence rate of β-scission reactions in the product significantly increased, lowering the molecular weight of mPAO and the product viscosity. Moreover, excessive cracking reactions may lead to an expanded molecular weight distribution, a lower flash point, and the diminished thermal stability of the base oil, thereby impacting the overall performance of the lubricant. Thus, the TPPT-modified mPAO product with a sulfur content of 0.389% (T-mPAO) was chosen for subsequent comparative study, owing to its fewer cracking products and anti-oxidation ability. To further compare the differences in performance with the sample dissolved in the PAO base oil, an mPAO base oil containing 1.5% TPPT was prepared at room temperature (saturation concentration of 1.55%, marked as A-mPAO).

The physical and chemical characterizations of mPAO, A-mPAO, and T-mPAO are shown in Table 1. The viscosity of the modified T-mPAO product significantly increased during the synthesis process due to the predominant alkylation reaction, while a lower viscosity index was observed. This can be attributed to the incorporation of more aromatic compounds into the long-chain structure, which minimally contributed to the viscosity index. Furthermore, there was a slight reduction in the flash point due to the presence of trace cracking reactions. The bromide value of mPAO was used to estimate the concentration of unsaturated double bonds in the reaction starting material as approximately 11.92 mmol/100 g. Assuming that all unsaturated double bonds in mPAO underwent a full single-alkylation reaction with TPPT, the theoretical maximum sulfur content in the product would reach 0.38%. However, the reaction results indicated that the maximum sulfur content can reach 0.48%, suggesting that unsaturated double bonds may have formed through β-cleavage during the reaction. These newly formed unsaturated double bonds further reacted with excess TPPT, increasing the sulfur content of the product. Despite introducing the high polarity compound TPPT into these modified products, the aniline point of the mPAO starting material remained unchanged at 170 °C and above.

### 3.4. Fourier Transform Infrared Spectra of mPAO and T-mPAO

**Figure 6:**
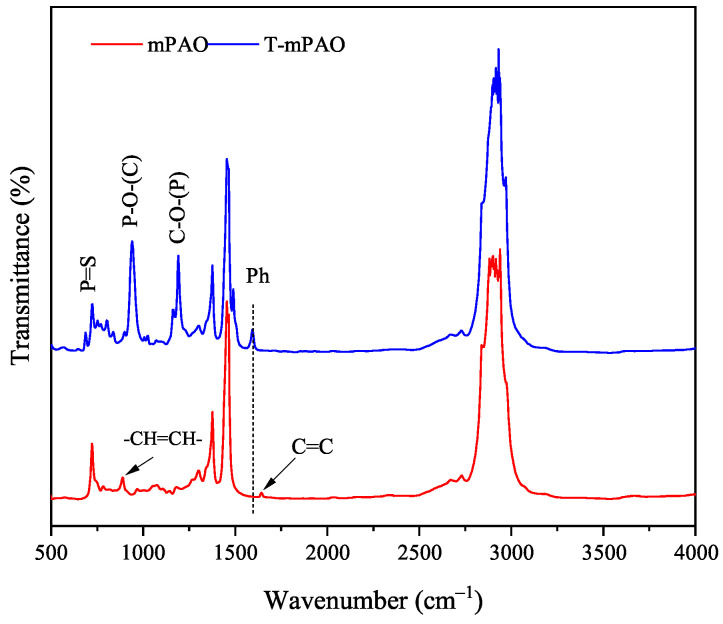
Fourier transform infrared spectra of mPAO and T-mPAO.

The FT-IR spectra of mPAO and T-mPAO are shown in Figure 6. In mPAO, the characteristic peaks at 1643 and 888 cm^−1^ were respectively attributed to the C=C stretching vibration of terminal olefins and the olefin C–H out-of-plane bending vibration. In T-mPAO, both characteristic peaks disappeared, and four new peaks appeared. Specifically, the peak at 1593 cm^−1^ was attributed to the aromatic structure, while the peak at 1192 cm^−1^ was ascribed to the stretching vibration of C–O bonds. Furthermore, the characteristic peak at 940 cm^−1^ corresponded to the stretching vibration of the P–O bond in TPPT, while the characteristic peak of the S=P bond was identified at 688 cm^−1^ [12]. These spectral changes indicated that the unsaturated double bond in mPAO had undergone an alkylation reaction with TPPT, successfully introducing TPPT into the molecular structure of the mPAO base oil.

### 3.5. Nuclear Magnetic Resonance Spectra of mPAO and T-mPAO

**Figure 7:**
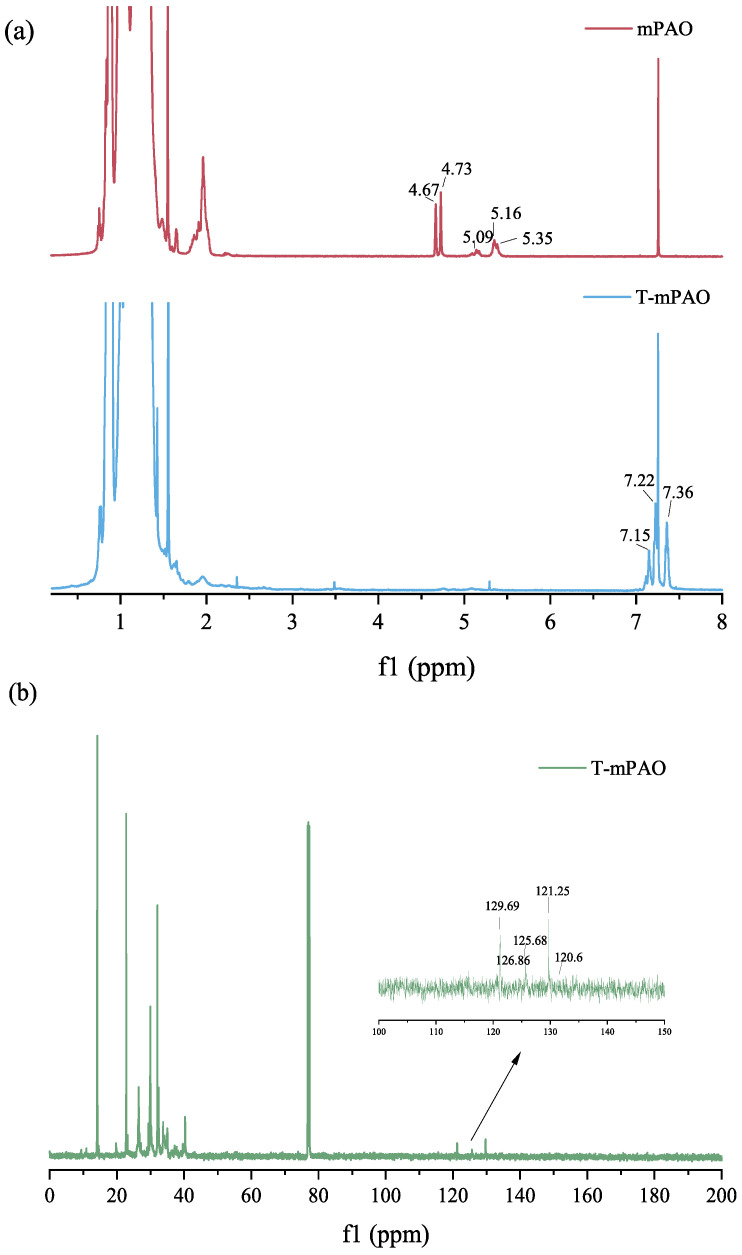
(**a**) ^1^H NMR and (**b**) ^13^C NMR of mPAO and T-mPAO.

In the preparation of polyalphaolefin with metallocene catalysts, three types of unsaturated double bonds (vinylidene, tri-substituted vinylene, and vinylene) formed during chain termination due to β-H elimination or rearrangement [31]. The ^1^H and ^13^C NMR spectra of mPAO and T-mPAO are shown in Figure 7. In the ^1^H NMR spectrum of mPAO, the pair of double peaks at 4.67 and 4.73 ppm belonged to vinylidene. The peaks at 5.0–5.2 ppm were assigned to tri-substituted vinylene, formed by rearrangement during β-H elimination, while those at 5.3–5.5 ppm were assigned to vinylene. In the ^1^H NMR spectrum of T-mPAO, almost no chemical shifts corresponding to unsaturated double bonds were observed in the 4.6–5.5 ppm range, indicating that the double bond was almost completely alkylated and consumed. Additionally, characteristic benzene signals in TPPT were observed at 7.22 and 7.36 ppm, while the double peak signal at 7.15 ppm was attributed to protons on the benzene ring in TPPT that was partially substituted by mPAO [12]. Two weak characteristic signals at 120.6 and 126.8 ppm were found in the ^13^C NMR spectrum of T-mPAO, which were assigned to substituted benzene with an alkyl group at the para position [12]. The peaks detected at 129.7, 125.7, and 121.5 ppm were ascribed to the unsubstituted benzene ring in TPPT. NMR analysis confirmed the successful alkylation reaction between mPAO and TPPT. Furthermore, the unsaturated double bond present in mPAO had been almost completely converted.

### 3.6. Molecular Weight of mPAO and Its Derivatives

The molecular weight distributions of mPAO and modified mPAO produced at 90 (90-mPAO) and 110 °C (110-mPAO) are shown in Table 2. The alkylation of mPAO with TPPT affected both its molecular weight and molecular weight distribution. The molecular weight distributions of the alkylation products were compared at different reaction temperatures. The modified mPAO had a significantly lower molecular weight and a broader molecular weight distribution than the mPAO raw material used in the reaction. These results suggested that cracking reactions occurred during the alkylation process, leading to the generation of smaller molecular substances that contributed to the broader molecular weight distribution for the base oil. Therefore, optimizing the reaction temperature is crucial for minimizing side reactions and enhancing the quality of the base oil.

### 3.7. Thermal Oxidative Stability

**Figure 8:**
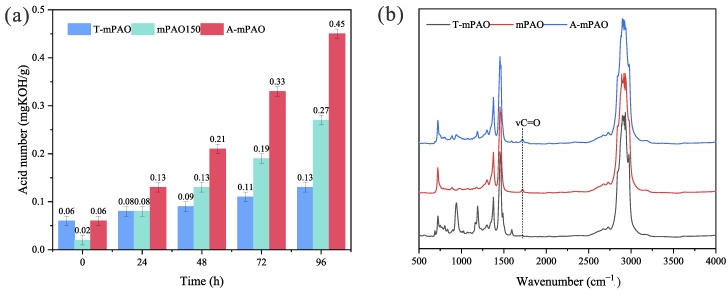
(**a**) Acid number and (**b**) FT-IR spectra of mPAO, A-mPAO, and T-mPAO after 96-h oven oxidation.

The anti-oxidation properties of the base oil have an important impact on the service life of the lubricating oil. Therefore, it was necessary to evaluate the anti-oxidation ability of chemically modified mPAO. The anti-oxidation ability of T-mPAO was evaluated by PDSC and oven oxidation (Table 3 and Figure 8). Compared with the IOT (178 °C) of mPAO, the IOT of modified T-mPAO (210 °C) was significantly improved, but The IOT of A-mPAO (183 °C) is only 5 °C higher than the IOT of mPAO.

To further evaluate the anti-oxidation performance of alkylated modified mPAO, an oven oxidation test was conducted at 150 °C with copper and iron as catalysts. The samples were subjected to accelerated oxidation treatment, and the acid number of each sample was measured every 24 h. As the oxidation test progressed, the acid numbers of all samples increased. The rate of increase in the acid number of A-mPAO was the fastest, followed by that of mPAO 150, with modified mPAO (T-mPAO) having the slowest rate of increase. After the 96-h oxidation experiment, the order of acid numbers was as follows: A-mPAO > mPAO 150 > T-mPAO. FT-IR analysis of the oxidized samples revealed a new absorption peak at 1720 cm^−1^ in the spectra of all tested samples, which was attributed to the vibration of the carbonyl (C=O) group produced by oil oxidation. Moreover, the intensity of this absorption peak for the samples followed the same order as the acid number. The above evaluation demonstrated that modification by TPPT alkylation can enhance the anti-oxidation capability of mPAO, and a high sulfur content can further augment this anti-oxidation ability. The increase in the acid value of base oil is usually caused by oxidation of the base oil, and the unsaturated double bonds in the base oil are easily oxidized into organic carboxylic acids, causing the acid value of the base oil to increase rapidly. Because both mPAO and A-mPAO contain alkene double bonds, their acid value increases more rapidly than T-mPAO, For A-mPAO, TPPT dissolved in mPAO is prone to hydrolysis reactions with moisture in air to form phenol, which can initiate further oxidation reactions, and is thus not an effective antioxidant [32]. Consequently, the acid value of A-mPAO increases at a significantly higher rate. In contrast, T-mPAO contains virtually no unsaturated double bonds. Furthermore, during thermal oxidation, alkylated TPPT adheres to the metal surface through sulfur or oxygen adsorption, with the initial cleavage of the S=P double bond [32]. The positively charged phosphorus can capture hydrocarbon proxy radicals produced from the reaction between oxygen and free radicals in the base oil, thus forming phosphate esters and interrupting the chain reaction of free radical oxidation. Therefore, T-mPAO has the smallest oxidation rate.

**Figure 9:**
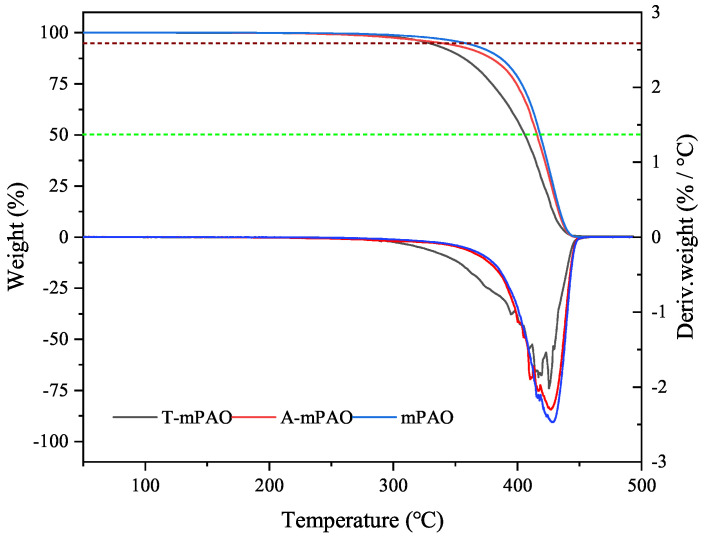
Thermogravimetric and differential thermogravimetric curves of mPAO, A-mPAO, and T-mPAO.

The TGA and differential TGA (DTG) curves of mPAO, A-mPAO, and T-mPAO are depicted in Figure 9. The 5% weight loss temperatures for the three samples were 357 °C (mPAO), 337 °C (A-mPAO), and 325 °C (T-mPAO). When the temperature was further increased, two smaller peaks were observed at 395 and 408 °C on the DTG curve of T-mPAO, indicating two different decomposition processes for two specific compounds at these temperature points. This finding was consistent with the generation of various alkylation products as described in the TPPT reaction mechanism. Meanwhile, a new peak was observed at 410 °C for A-mPAO, which may be the decomposition of a substance related to the dissolved TPPT in mPAO. The 50% weight loss temperatures of mPAO, A-mPAO, and T-mPAO were 418, 415, and 405 °C, respectively. Besides alkylation reactions, β-cleavage reactions also occurred during the modification process, yielding small molecular substances that reduced the thermal stability of mPAO compared to the original PAO. Therefore, it is crucial to control the reaction conditions to maintain a level of thermal stability for the base oil.

### 3.8. Tribological Results

#### 3.8.1. Tribological Performance of Point-to-Point Contact on a Four-Ball Friction Tester

**Figure 10:**
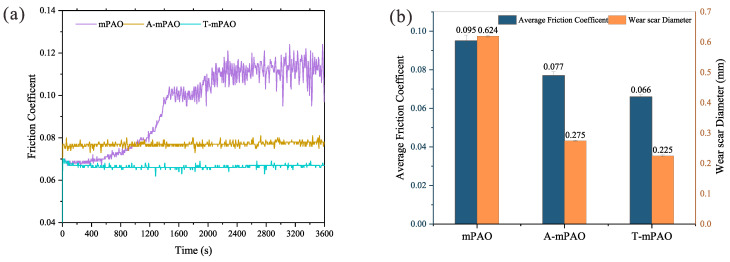
(**a**) Friction coefficient curve, (**b**) wear track diameter and average friction coefficient of mPAO, A-mPAO and T-mPAO in constant-speed friction test on four ball tester at 196 N and 1200 rpm.

The friction coefficient curve, wear track diameter, and average friction coefficient of mPAO, A-mPAO, and T-mPAO in a constant-speed friction test at 196 N and 1200 rpm are presented in Figure 10. A-mPAO and T-mPAO exhibited a brief “run-in” and rapidly achieved a steady state with a consistent friction coefficient. Unlike A-mPAO and T-mPAO, mPAO showed an increasing friction coefficient throughout the test, with a noticeable fluctuation in the later stages. Steady-state friction coefficients are typically divided into four types according to the friction mark: (Type A) the friction coefficient remained essentially constant during the steady-state period; (Type B) the friction coefficient gradually decreased during the test; (Type C) the friction coefficient gradually increased during the test; and (Type D) the friction coefficient fluctuated throughout the test [33]. The friction curves of T-mPAO and A-mPAO were Type A, which is ideal for the lubrication process. However, the friction curve of the mPAO base oil was a combination of Type C and D, which is an undesirable non-ideal state. Compared to mPAO, the average friction coefficient of A-mPAO (0.077) was lower, and the diameter of the wear track was 55% smaller. T-mPAO exhibited the lowest average friction coefficient (0.066) and the smallest wear track diameter (0.225 mm) among the samples, exhibiting a 64% reduction in the wear track diameter compared to the mPAO base oil (0.624 mm). The results indicated that following modification with TPPT, mPAO exhibits enhanced lubricating and anti-wear characteristics. In comparison to mPAO and A-mPAO, T-mPAO possesses the highest concentrations of sulfur and phosphorus, which are uniformly grafted onto the extended main chain of mPAO. In the friction testing, T-mPAO is more readily able to reach the metal surface and rapidly establish a chemical film. When the friction pair attains a boundary lubrication state, the tribofilm can effectively lower the friction coefficient and reduce wear on the contact surfaces of the friction pair, thereby protecting their contact surface.

**Figure 11:**
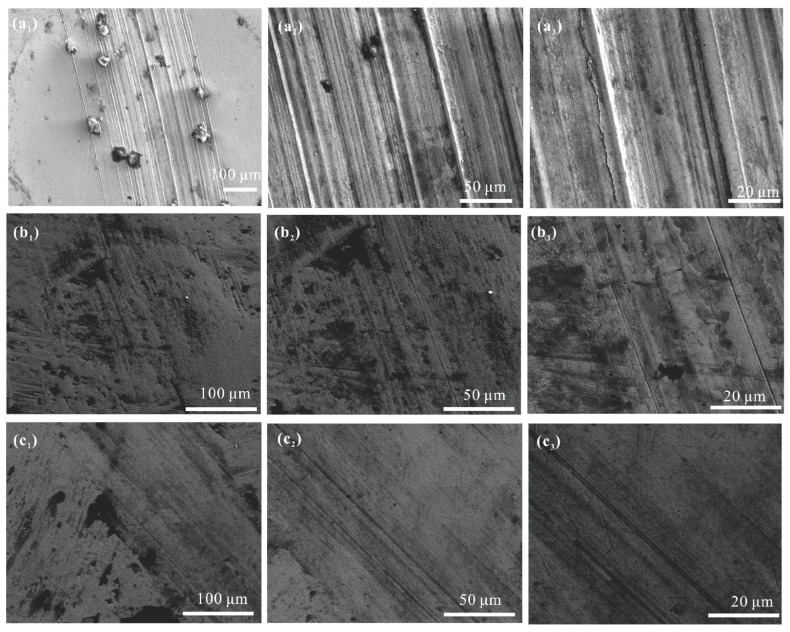
Wear track morphologies of (**a1**–**a3**) mPAO, (**b1**–**b3**) A-mPAO, and (**c1**–**c3**) T-mPAO at different magnifications.

The worn surface morphologies of the studied samples are shown in Figure 11. A high density of closely spaced furrows and noticeable wear debris were observed in the mPAO wear marks, indicating severe abrasive wear during the friction process. Compared to A-mPAO, the wear marks on T-mPAO were significantly smaller, and only shallow and sparse scratches were produced in the sliding direction. The experimental results show that after chemical modification by TPPT, the modified mPAO base oil could form a friction-reducing protective film on the lubrication interface during the friction test, significantly reducing friction and wear. This demonstrated the excellent anti-wear properties of mPAO after modification compared to the PAO base oil containing TPPT.

#### 3.8.2. Tribological Performance of Point-on-Flat and Line-on-Flat Contact on a TE77 Reciprocating Friction Tester

**Figure 12:**
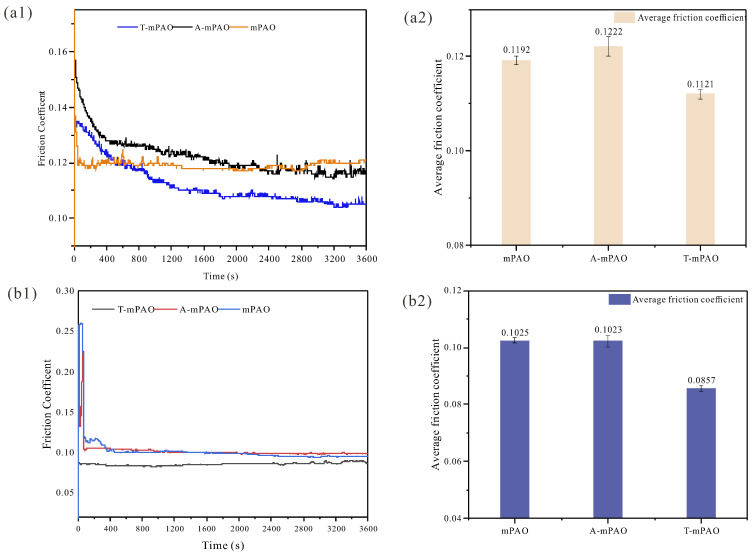
Friction coefficient curves and average friction coefficients of mPAO, A-mPAO, and T-mPAO in the point-on-flat (**a1**,**a2**) and line-on-flat contact modes (**b1**,**b2**).

The point-on-flat and line-on-flat contact modes of the samples were studied using a TE77 reciprocating friction tester. The tribological properties displayed in the reciprocating sliding friction mode differed from those in the four-ball friction test. The friction coefficient curves and average friction coefficient are shown in Figure 12 for both test modes. In the point-on-flat mode, all three samples quickly reached a steady state, with the average friction coefficient of T-mPAO being the smallest, showing a certain degree of antifriction effect. However, the friction coefficient of A-mPAO was almost the same as that of the mPAO base oil, indicating that TPPT did not have an antifriction effect in this mode. In the line-on-flat mode, the friction coefficient of T-mPAO gradually decreased as the test progressed and had the lowest average friction coefficient, while the average friction coefficients of A-mPAO and the mPAO base oil were similar to those in the point-on-flat mode. This suggested that T-mPAO had better anti-friction capabilities than A-mPAO.

**Figure 13:**
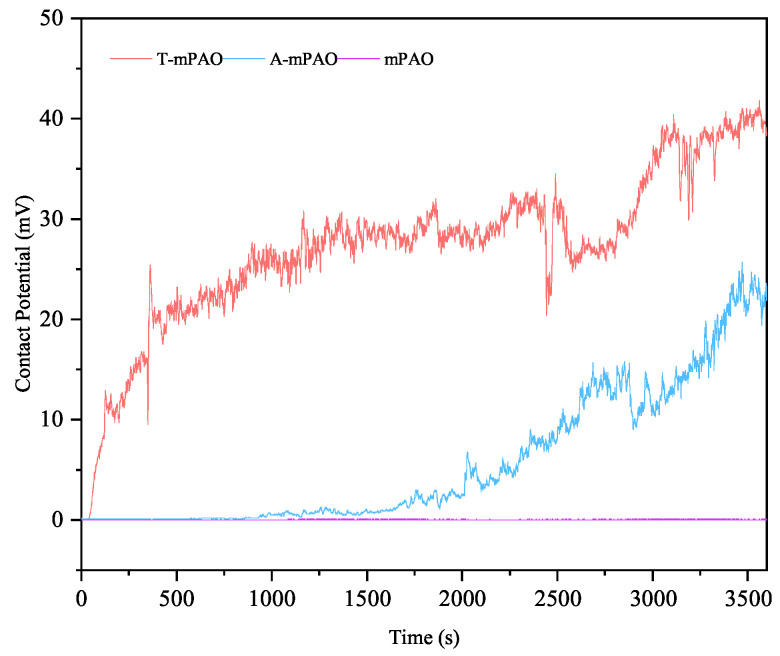
The contact potential curves of mPAO, A-mPAO, and T-mPAO in the point-on-flat contact mode.

Analysis of the contact potential profile (Figure 13) revealed that T-mPAO exhibited a rapid initial increase in potential, which continued for the duration of the experiment. The polar groups in the modified mPAO molecular structure can rapidly adsorb onto the worn surface to form a protective oil film layer, reducing friction while enhancing the contact potential. As the test progressed, a layer of chemical film containing sulfur and phosphorus was generated on the worn surface, further reducing the friction coefficient [34], and the contact potential continued to increase. Meanwhile, A-mPAO gradually formed a chemical protective film, leading to a lower coefficient of friction and an increased contact potential. The mPAO base oil, due to its non-polar properties, was unable to form an effective oil film and chemical film during boundary lubrication, so the two friction pairs came into direct contact without generating any electric potential.

**Figure 14:**
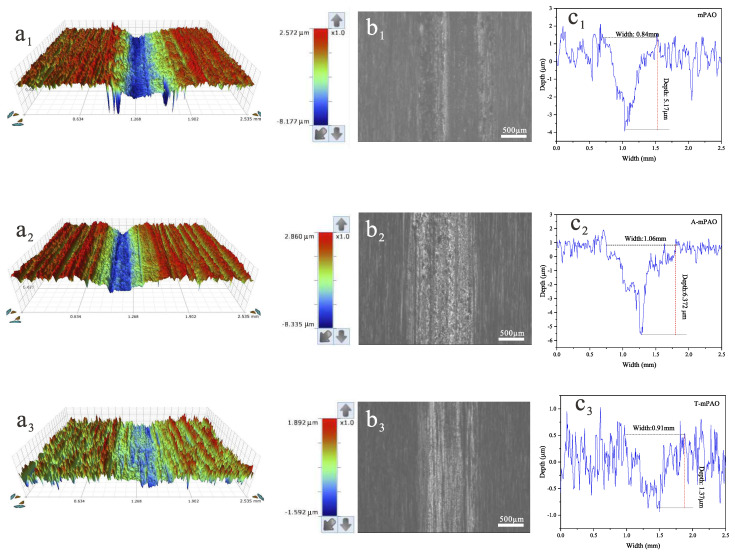
Three-dimensional shape, micrographs and size of wear marks of steel plate after TE77 point on flat friction tests (**a1**,**b1**,**c1**): mPAO, (**a2**,**b2**,**c2**): A-mPAO, (**a3**,**b3**,**c3**): T-mPAO.

**Figure 15:**
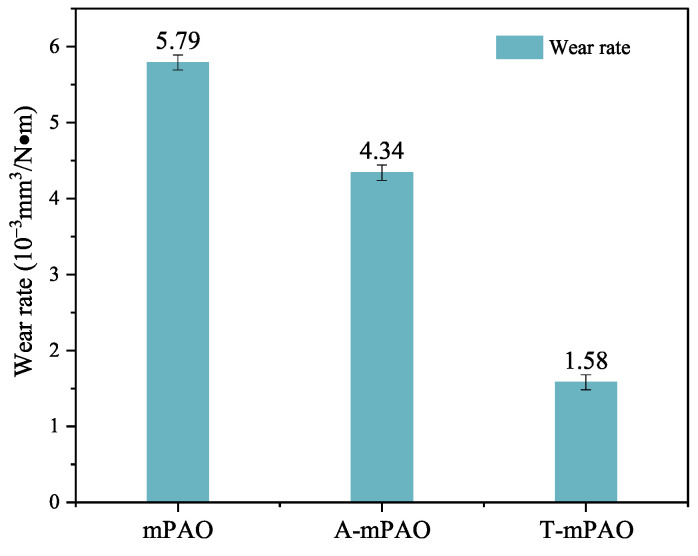
Wear rates of steel plates lubricated with mPAO, A-mPAO, and T-mPAO after point-on-flat contact at 392 N, 5 Hz, and 75 °C on a TE77 tester.

The surface morphologies of the steel plates after the TE77 friction tests were characterized by white light interferometry. Figure 14 depicts the three-dimensional profiles of the worn surfaces after lubrication with mPAO, A-mPAO, and T-mPAO. After the friction experiment on mPAO and A-mPAO, similar wear marks appeared, with a deep wear depth (5.17–6.37 μm) and rough surface. However, the depth of the wear marks of T-mPAO (1.37 μm) was much lower than those of mPAO and A-mPAO. Based on these measurements, the wear rates of the samples were calculated (Figure 15). Compared with mPAO, the wear rate of A-mPAO decreased by 25%, while the wear rate of T-mPAO was reduced significantly by 72.7%. These results indicated that mPAO modified by TPPT had excellent anti-wear properties, which was consistent with the results of the four-ball friction test.

#### 3.8.3. Extreme Pressure Performance of mPAO, A-mPAO, and T-mPAO

Triphenyl phosphorothioate, which contains sulfur and phosphorus elements, is usually used as an anti-wear additive in lubricating oil formulations for use under extreme pressure. The effects of modification on the extreme pressure performance of the base oil were examined, and the results are shown in Table 4. After modification, the extreme pressure performance of T-mPAO improved significantly, with a *P*_*B*_ (238 kg) 2.5 fold that of mPAO (94 kg) and almost twice that of A-mPAO. Similarly, the *P*_*D*_ also increased from 126 kg (mPAO) to 250 kg (T-mPAO), showing excellent extreme pressure performance. This improvement in comparison may be due to the absence of active elements in mPAO, which cannot form an effective protective film, leading to rapid sintering. Almost every mPAO molecule in T-mPAO contains a polar sulfur atom and a phosphorus-rich TPPT group, and the levels of phosphorus and sulfur are higher than those in A-mPAO. This makes them easier to adsorb onto metallic surfaces, leading to the formation of oil and chemical films through friction chemistry, significantly enhancing the load-bearing capacity of the oil while preventing direct contact with the metal. During the friction process, the chemical film is constantly consumed, but the tendency to easily adsorb also allows the friction chemical film to be quickly replenished, thus delaying the sintering of the metals [8].

#### 3.8.4. Elemental Analysis of Worn Surfaces

**Figure 16:**
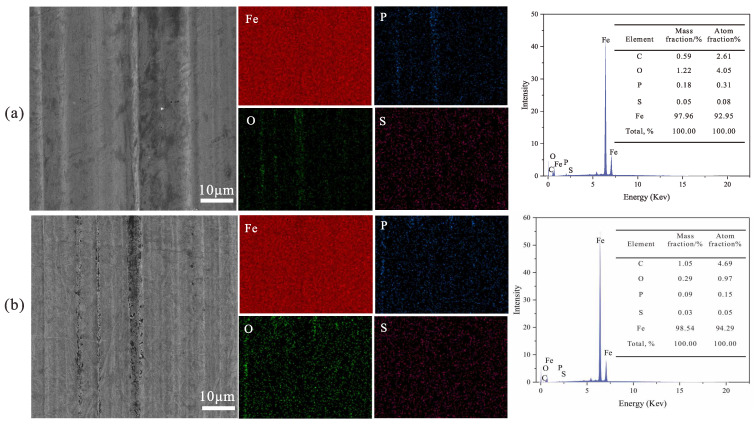
SEM and EDS images of the wear spot surfaces of (**a**) T-mPAO and (**b**) A-mPAO after point-on-point contact tests at 196 N, and 75 °C on a four ball tester.

**Figure 17:**
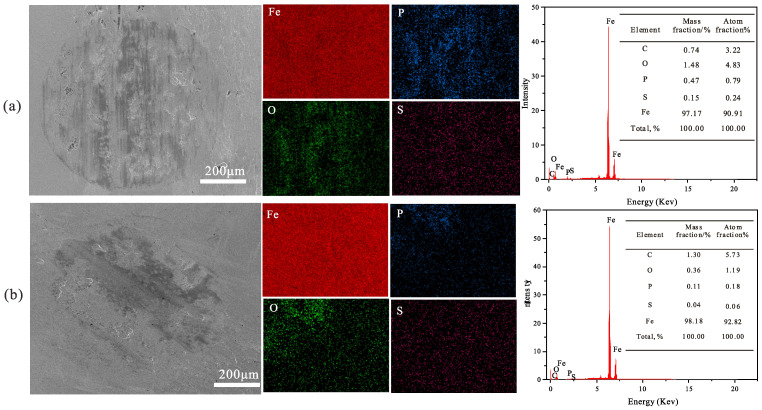
SEM and EDS images of the wear spot surfaces of (**a**) T-mPAO and (**b**) A-mPAO after point-on-flat contact tests at 392 N, 5 Hz, and 75 °C on a TE77 tester.

The element types and contents of the wear area on the upper test ball surface in the four ball friction test and reciprocating sliding test were analyzed by energy dispersive spectroscopy (EDS) on an X-ray photoelectron spectrometer (Figure 16 and Figure 17). SEM images revealed that the wear track diameters of both materials were comparable in reciprocating sliding test; however, those of T-mPAO exhibited distinct and uniform circular shapes with a relatively smooth surface, while the wear tracks of A-mPAO were irregular in shape with a rough surface marked by noticeable scratches and a discernible trailing edge. EDS analysis of the wear areas of both materials indicated that both worn surfaces contained S, P, and O with uniform distribution and no obvious clustering. In the four ball friction test, the sulfur and phosphorus content on the worn surface is lower than that of reciprocating sliding test. Moreover, the contents of S and P in T-mPAO were significantly higher than that of A-mPAO in all test. Sulfur and phosphorus react with iron under high heat and pressure in the presence of oxygen to form compounds such as sulfuric acid iron and phosphoric acid iron, creating a protective layer on the friction surface that reduces the wear of the friction pair [35]. The higher sulfur and phosphorus contents indicate the formation of a more extensive and dense friction film. This aligns with the aforementioned experimental results, regardless of whether T-mPAO performed well in the four-ball or TE77 reciprocating friction tests.

**Figure 18:**
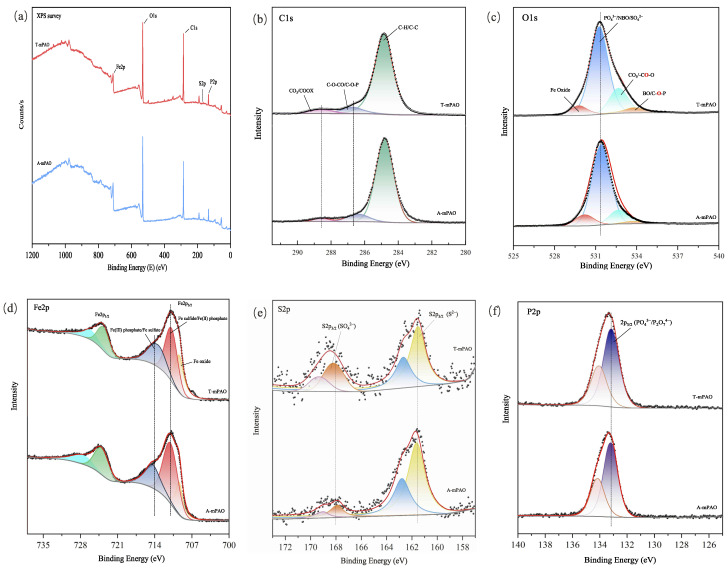
High-resolution XPS (**a**) survey, (**b**) C 1s, (**c**) O 1s, (**d**) Fe 2p, (**e**) P 2p, and (**f**) S2p spectra of wear marks on the upper steel ball at 392 N, 5 Hz, and 75 °C on a TE77 tester.

XPS analysis of the worn surface of the upper steel ball in the point-on-flat contact friction test was conducted, revealing the elemental states (Figure 18). The survey revealed the presence of carbon (C 1s, 285 eV), oxygen (O 1s, 532 eV), iron (Fe 2p_3/2_, 712 eV), sulfur (S 2p_3/2_, 168 eV), and phosphorus (P 2p_3/2_, 134 eV) in the wear area [36]. The C 1s spectrum shows a strong signal at 284.8 eV, which belonged to aliphatic carbon (C–C, C–H), and weaker signals at 286.6 and 288.5 eV that respectively correspond to C–O and CO_3_/COOX [37]. The high-resolution O 1s spectrum was resolved into four signals. The low-binding energy peak at 529.7 eV was attributed to iron oxides, the main peak at 531.3 eV originated from non-bridging oxygen (NBO, e.g., P=O) in phosphates and sulfates (SO_4_^2−^); the peak at 532.7 eV belonged to the carbonate/carbonyl oxygen in esters and ketones, and the peak at 533.7 eV corresponded to the bridging oxygen (BO) in ester compounds [38].

The Fe 2p spectrum shows two main peaks at 710.8 eV (Fe 2p_3/2_) and 726 eV (Fe 2p_1/2_, spin-orbit splitting). Only the Fe 2p_3/2_ signal was analyzed, with the main components being FeO(II) at 709.4 eV and ferrous phosphate/sulfide at 710.8 eV. The peak at 713.5 eV is confirmed as one of the main components of the friction film, corresponding to ferric phosphate/sulfide [39].

The S 2p spectrum showed two main peaks, each with S 2p_3/2_ and S 2p_1/2_ components, with signals at 161.5 and 168.1 eV corresponding to ferrous sulfide and sulfate (SO_4_^2−^), respectively [40]. For T-mPAO and A-mPAO, the chemical states and relative contents of Fe, O, and C in the wear spot were basically consistent, but there was a significant difference in the distribution of sulfur chemical states. The relative content of sulfate in T-mPAO is higher than that in A-mPAO, as a higher content of sulfur in T-mPAO was more easily adsorbed onto the friction surface and further oxidized to sulfate.

The P 2p spectrum showed two peaks at 2p_3/2_ and 2p_1/2_ due to spin-orbit splitting, with the peak at 133.2 eV corresponding to the phosphate group. No characteristic peaks of long-chain polyphosphates were detected in the wear areas of T-mPAO and A-mPAO.

#### 3.8.5. Lubrication Mechanism

**Figure 19:**
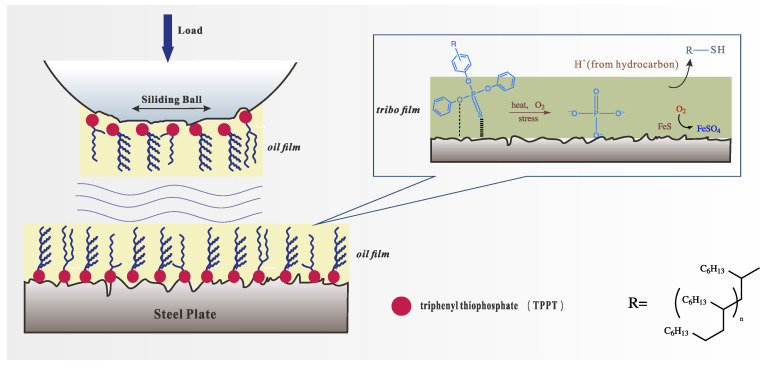
Lubrication mechanistic diagram of TPPT-modified mPAO on the surface of the friction pair.

Based on the SEM, EDS, and XPS analyses, the lubrication mechanism of TPPT-modified mPAO was proposed (Figure 19). T-mPAO quickly adhered to the surface of the friction pair through its polar TPPT group in its molecular structure, forming a dense physical–chemical adhesion film. Meanwhile, the PAO long main chain formed an oil film layer, providing further protection for the friction pair. As the friction test proceeded, the S=P bonds of the TPPT groups adsorbed on the friction pair surface were first broken under the action of shear force, friction heat, and oxygen [28]. These groups reacted with Fe to form iron sulfide, which was further oxidized to ferric sulfate under the action of oxygen. A portion of the sulfur may have also reacted with fragmented alkyl groups to produce thiol compounds that were soluble in the base oil [12]. An analysis of the P 2p spectrum of phosphorus did not reveal any characteristic peaks associated with long-chain polyphosphates; only PO_4_^3−^ was detected. This may be attributed to the insufficient time to produce polyphosphates during the 1-h friction test. The generated phosphate and sulfate formed a chemical film that continued to protect against wear and tear. As the friction test progressed, the chemical film was continuously stripped and consumed. Since the basic structure of each T-mPAO molecule contained a TPPT group, it could more efficiently adsorb to the surface of the friction pair for replenishment than A-mPAO, resulting in its superior anti-wear performance.

## 4. Conclusions

Under the catalysis of trifluoromethanesulfonic acid, the chemical modification of mPAO with high viscosity containing terminal olefins was achieved through alkylation with triphenyl phosphorothioate. and the preliminary reaction mechanism was interpreted. The antioxidant and wear-resistant properties of chemically modified mPAO and mPAO with dissolved TPPT were compared in this study. Through the analysis of the morphology and element state via friction tests, the lubrication mechanism of TPPT-modified mPAO was proposed and the following conclusions were drawn:

(1) TfOH is an efficient alkylating modification catalyst that can enhance the content of sulfur and phosphorus in the product by increasing the reaction temperature and catalyst dosage. During the reaction process, alkylation and β-cleavage reactions occurred simultaneously. The success of the alkylation reaction was confirmed by NMR and FTIR analyses, indicating that almost all the double bonds in the starting material were successfully converted into saturated molecules.
(2) The PDSC and oven oxidation experiments confirmed that the TPPT-modified mPAO had significantly improved oxidation resistance compared to mPAO.
(3) In the four-ball and TE77 reciprocating friction tests, TPPT-modified mPAO demonstrated superior wear resistance. SEM, EDS, and XPS analyses indicated that during the friction test, a chemical film of wear-resistant ferro-sulfate and ferro-phosphate was generated on the friction surfaces.

The chemical modification of mPAO has imparted additive-like properties to the base oil. This novel synthetic process simplifies the design of lubricant formulations, reducing the need for additives, enabling it to adapt to more stringent operating conditions. Except the introduction of anti-wear and friction-reducing functional groups, it is also possible to introduce functional groups that enhance dispersibility, reduce sludge formation, and provide anti-corrosion properties, thereby expanding the application scenarios of mPAO base oil.

## Figures and Tables

**Table 1:** Physicochemical properties of mPAO, A-mPAO and T-mPAO.

Pramater	mPAO	T-mPAO ^1^	A-mPAO
Kinematic viscosity, mm^2^/s			
40 °C	1622	2283	1618
100 °C	150.2	186.6	149.2
VI	205	203	204
Flash point/°C	285	280	285
Pour point/°C	−33	−27	−33
Acid number, mg KOH/g	0.02	0.06	0.06
Bromine number, gBr/100 g	1.908	/	/
Sulphur content, wt%	0	0.389	0.150
Aniline point, °C	>170	>170	>170

^1^ Reaction conditions: TfOH: 3 mL, reaction temperature: 70 °C, reaction time: 12 h, mPAO/TPPT = 19.4.

**Table 2:** Molecular weight of mPAO and modified mPAO produced at 90 and 110 °C.

Sample	Mz (Daltons)	Mn (Daltons)	Mw (Daltons)	Mw/Mn
mPAO	11,147	3439	6578	1.89
110-mPAO	11,189	3254	6680	2.05
90-mPAO	11,175	3530	6920	1.96
T-mPAO	11,772	3798	7316	1.92

**Table 3:** Initial oxidation temperature of mPAO, A-mPAO, and T-mPAO.

Parameter	mPAO	A-mPAO	T-mPAO
IOT, °C	178	183	210

**Table 4:** *P*_*B*_ and *P*_*D*_ of mPAO, A-mPAO, and T-mPAO.

Sample	*P*_*B*_/kg	*P*_*D*_/kg
mPAO	94	126
A-mPAO	171	200
T-mPAO	238	250

## Data Availability

The original contributions presented in the study are included in the article, further inquiries can be directed to the corresponding authors.
